# Therapeutic Power of Date Fruit (*Phoenix dactylifera* L.): A Nutrient‐Rich Superfood for Holistic Health and Disease Prevention

**DOI:** 10.1002/fsn3.70896

**Published:** 2025-09-07

**Authors:** Kanza Aziz Awan, Sanabil Yaqoob, Iahtisham Ul‐Haq, Hiba Naveed, Aysha Imtiaz, Amal Shaukat, Waleed Sultan, Jian‐Ya Qian, Esther Ugo Alum, Qing Shen

**Affiliations:** ^1^ Department of Food and Nutritional Sciences, Faculty of Science and Technology University of Central Punjab Lahore Pakistan; ^2^ Panvascular Diseases Research Center, Quzhou People's Hospital The Quzhou Affiliated Hospital of Wenzhou Medical University Quzhou China; ^3^ Laboratory of Food Nutrition and Clinical Research, Institute of Seafood Zhejiang Gongshang University Hangzhou China; ^4^ Kauser Abdulla Malik School of Life Sciences Forman Christian College (A Chartered University) Lahore Pakistan; ^5^ College of Food Science and Engineering Yangzhou University Yangzhou Jiangsu China; ^6^ Department of Research and Publications Kampala International University Kampala Uganda

**Keywords:** dates, extraction, functional foods, health benefits, oxidative stress cardiovascular disease

## Abstract

Date fruit ( *Phoenix dactylifera* L.) is a highly nutritious and therapeutic food with substantial potential to improve human health. This review emphasizes the nutritional and therapeutic traits of date fruit, focusing on its role as a functional food and dietary supplement. Rich in essential nutrients, antioxidants, and bioactive moieties, date fruit has been shown to provide numerous health benefits. These include managing metabolic disorders such as dyslipidemia, hyperglycemia, liver toxicity, kidney toxicity, obesity, and cardiovascular diseases. Studies exhibit that regular date consumption can contribute to the prevention of chronic illnesses and promote overall health and well‐being. However, while research on individual bioactive compounds has been extensive, the full biological effects of the fruit, particularly in synergistic contexts, are not yet fully understood. This review consolidates and critically evaluates recent in vitro, in vivo, and clinical findings on date fruit's bioactive substances, particularly flavonoids, phenolic acids, and dietary fiber, and their role in modulating metabolic disease. Unlike previous studies, this review highlights the specific models and effective dosages: for example, animal studies using 300–1000 mg/kg/day of date extracts showed improvements in lipid profiles and antioxidant enzyme activity (SOD, CAT, GST), while in vitro assays at 60–600 μg/mL demonstrated anti‐inflammatory and anti‐apoptotic mechanisms via NF‐κB inhibition and cytokine downregulation (IL‐6, COX‐2, TNF‐α). Furthermore, HPLC‐ESI‐MS profiling showed cultivar‐specific differences in polyphenol content and antioxidant potential. The paper also explores lesser‐studied effects such as neuroprotection, immunomodulation, and antitumor activity. By integrating dosage‐specific mechanistic insights and model‐based outcomes, this review offers a more targeted framework for developing functional foods and nutraceuticals from date fruit and highlights the urgent need for further clinical trials to validate these findings and optimize therapeutic applications.

## Graphical abstract

This review highlights the nutritional and therapeutic attributes of date fruit, focusing on its role as a functional food and dietary supplement. This review consolidates existing findings and underscores the importance of further investigation into the therapeutic potential of date fruit, particularly in the development of functional foods and nutraceuticals.

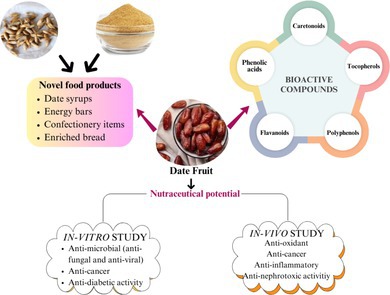

## 1 Introduction

The socio‐economic demographic transitions in Asia have been significantly influenced by urbanization, globalization, and westernization over recent decades. These transformations have reshaped living patterns and dietary practices across the region (Brandtzaeg 2009). Epidemiological investigations indicate a rising incidence of chronic diseases in various populations, particularly in Asia. The most frequently reported conditions include CVDs, high sugar, blood pressure, osteoporosis, obesity, cancer, and high cholesterol (Naveed et al. 2024). These changes are driven by the rapid evolution of food consumption patterns and lifestyle adaptations. This contemporary diet is often characterized by an increased intake of high‐calorie, fat‐rich, sugar‐laden snacks and confections, which are emerging as major contributors to various health risks (Pingali 2007; Reardon et al. 2014).

The date palm (*Phoenix dactylifera L*.) is a fruit with great nutritional value that can significantly improve human health (Awan et al. 2025). It is widely known as the “tree of life” and the “King of Oasis” because of its capacity to flourish in dry climates and support the local populace. The Arecaceae family includes the date palm, which is a dioecious plant with separate male and female parts. It holds significant commercial value and is extensively utilized in various food applications. Although date palms are grown worldwide, certain weather conditions are necessary for the best fruit production (Baliga et al. 2011; Zaid and Arias‐Jimenez 2002). The date fruit market is projected to continue its growth, with estimates suggesting it will reach USD 13,500 million by 2023. The top six producing nations, including Pakistan, which is the fifth‐largest producer with an annual output of 650,000 tons as of 2022, account for 75% of the world's total date production (FAO 2022). Date palms are cultivated globally, particularly in Southern Europe, the Middle East, North Africa, parts of Central and North America, and the Indo‐Pak region (Al‐Shahib and Marshall 2003).

The sugar and moisture content of dates determines their classification: semi‐dry dates have 13%–15% moisture, soft dates have 18%–24% moisture, and dry dates have 8%–10% moisture (Biglari et al. 2008). Soft dates can contain up to 80% invert sugars, whereas semi‐dry dates have 40% sucrose and 40% invert sugars (Bender 2009).

Different phases of growth are experienced by date fruits, which are important for reaching the required maturity for commercialization. These stages are defined by chemical and physical alterations. Fruit's color, texture, and sweetness are important markers of its ripeness and maturation (Al‐Farsi and Lee 2011). Each of the five maturation phases of date fruits, known as Kimri, Khalal, Hababouk, Tamr, and Rutab in Arabic, is crucial to this process.

The fruit experiences a drop in weight and moisture content and a rise in sugar content throughout the roughly 6‐week‐long Khalal stage. Depending on the cultivar, the fruit's color changes throughout this phase from green to yellow or red. The fruit reaches its maximum size and weight near the conclusion of the Khalal stage, and its texture becomes firm.

The Rutab stage follows the Khalal stage and typically lasts between 2 and 4 weeks, depending on varietal attributes, temperature, humidity, and irrigation conditions. During this phase, the fruit undergoes significant physiological changes; the apex begins to ripen, astringency decreases due to tannin polymerization, and the color darkens from yellow to brown. The moisture content drops moderately as water evaporates under warm, dry conditions, resulting in weight loss. Enzymatic activity increases, promoting the conversion of complex sugars (sucrose, starches) into simpler sugars (glucose and fructose), giving the fruit a soft and sweet texture.

The Tamr stage represents the fully ripened state, generally occurring under hot and arid conditions with prolonged exposure to sunlight and minimal rainfall. The fruit becomes darker (brown to black), firmer in texture, and has a substantially lower moisture content (often below 20%). This phase is characterized by maximum sugar concentration, especially monosaccharides, and increased protein levels, making the date highly caloric and shelf‐stable. However, moisture, lipid, and total weight decrease further due to continued water loss and biochemical shifts. These alterations are essential for post‐harvest handling, as fruits in the Tamr stage are more resistant to microbial spoilage and ideal for long‐term storage. In many cultivars, the outer skin clings to the inner flesh, causing wrinkles as the inner flesh shrinks (Baliga et al. 2011; Sahar et al. 2013). When date fruits are Tamr completely ripe or Rutab semi‐ripe, they are often eaten raw or with minimal preparation (Chandrasekaran and Bahkali 2013).

Given the various health‐promoting effects of date fruit, this study aims to provide an overview of the most recent research on the use of date fruit to treat conditions including oxidative stress, heart disease, liver disease, and kidney illness. The review will also include the process of removing beneficial bioactive substances from date fruit.

While several earlier reviews have acknowledged the nutritional and therapeutic benefits of *Phoenix dactylifera* L., most have lacked standardized dosage guidance, precise mechanistic insight, and cultivar‐level profiling. This review builds upon present literature by integrating the latest epidemiological, mechanistic, and metabolomic results. For instance, a 2025 meta‐analysis of randomized controlled trials demonstrated that date fruit consumption significantly reduces total cholesterol in patients with type 2 diabetes, though effects on LDL, HDL, and triglycerides were non‐significant (Mirghani and Alhowiti 2025). Comparative antioxidant profiling using UPLC‐QTOF‐MS across cultivars such as Ajwah, Sukkari, and Safawy revealed IC₅₀ values ranging from 103 to 177 μg/mL for DPPH assays and identified 22 distinct bioactive phenolics (Zihad et al. 2021). Additionally, LCESIMS metabolomic analyses across multiple cultivars elucidated extensive phenolic diversity and metabolite fingerprinting that previous reviews have not addressed (Bettaieb et al. 2023). By correlating these data with dosage‐specific in vivo and in vitro outcomes, this review provides a more integrative and translational framework linking biochemical activity (e.g., NF‐κB inhibition, cytokine downregulation, antioxidant enzyme upregulation) to disease‐specific applications and emphasizing the cultivar‐dependent distinctions.

This review uniquely contributes to the existing literature by providing updated and integrated insights on the biochemical, therapeutic, and technological potential of date fruit. Unlike previous reviews, it systematically categorizes health benefits based on specific bioactive moieties, especially polyphenols such as quercetin, rutin, ferulic acid, caffeic acid, and their mechanisms of action at the cellular and molecular level. For the first time, this paper emphasizes how these compounds interact with key signaling pathways like Nrf2–ARE (antioxidant defense), NF‐κB (inflammation), AMPK (metabolism), and SIRT1 (aging and mitochondrial health), supported by both in vitro and in vivo studies. Additionally, this review presents a new comparative table summarizing therapeutic dosages, biological models used, and observed health outcomes. It also explores the impact of thermal vs. non‐thermal processing on nutrient retention, offering practical implications for product development and functional food innovation. This multi‐dimensional approach strengthens the novelty of this manuscript within the scope of nutritional therapeutics and food science.

## 2 Nutrition and Health‐Promoting Effects of Dates

Proper nutrition is fundamental in both preventing and managing non‐communicable diseases (NCDs), playing a pivotal role in enhancing overall health and well‐being. These diseases are typically chronic, marked by a gradual progression over time. Often referred to as degenerative diseases, they lead to the slow deterioration of tissue structure and function. Various factors contribute to this, particularly lifestyle choices such as sedentary behavior, insufficient physical activity, and unhealthy dietary habits. Prolonged stress and anxiety, along with chronic undernutrition, can accelerate the aging and degeneration of mitochondria (Olaiya et al. 2016). To mitigate the occurrence and spread of NCDs, consuming fruits and vegetables is highly recommended (WHO 2020). The use of phytoceuticals in therapeutic practices is referred to as phytotherapy, which is a key aspect of herbal pharmacology. Since many foods are rich in various phytochemicals, the distinction between food and phytotherapy is often blurred. The active biological components found in these remedies can interact with multiple pharmacological targets. To validate their effectiveness, researchers conduct in vitro studies at cellular or tissue levels, as well as in vivo studies in animal models, to assess their preclinical and pharmacokinetic properties (Ramzan and Li 2015).

For centuries, therapeutic plants have played a crucial role in Arab‐Islamic medicine. Islamic teachings emphasize the consumption of fruits, vegetables, and herbs provided by God as a means of disease prevention and management. Over fourteen hundred years ago, Prophet Muhammad (PBUH) extolled the virtues of natural foods and their medicinal uses in various hadiths. These remedies are compiled in the book “Tib‐e‐Nabvi” (The Prophet's Medicine), assembled by several religious scholars. These principles underpin the contemporary functional food paradigm. The Prophet stated that Allah has created both diseases and their cures, except for aging, suggesting a belief in the availability of remedies for every ailment (Nagamia 2003; Ramzan and Li 2015).

### 2.1 Compositional Profiling of Date Fruit

The date fruit is composed of a seed and a fleshy pericarp, which constitutes approximately 85%–90% of the fruit's total weight. It is one of the rare plants where every part, including the flesh, pit, and leaves, can be effectively integrated into the food chain when processed correctly. In Persian traditional medicine, the water extracted from the spathe (the bract that encloses the inflorescence) of date palms, known as “Tarooneh,” is renowned for its rich content of volatile compounds and essential oils. Additionally, this extract is valued for its sedative properties (Hamedi et al. 2013; Mohamadi et al. 2014; Pourdarbani et al. 2012). Date pits are not only edible but also serve as a popular caffeine‐free coffee alternative. Furthermore, date pits are widely utilized in animal feed, offering an excellent source of dietary fiber, fatty acids, and minerals (Abdul Afiq et al. 2013; Al‐Farsi and Lee 2011; Alsaif et al. 2007; Nehdi et al. 2010). Similarly, other parts of the date palm, such as leaves and branches, are utilized in the artifact industries and paper mills. This comprehensive use of the date palm underscores the importance of mechanization and post‐harvest processing in. Maximizing the plant's economic and industrial potential. The region‐wise compositional profile of date fruit is given above in Table 1.

**TABLE 1:** Region‐wise compositional profile of date fruit table.

Region	Cultivar	Moisture (%)	Carbohydrates (%)	Protein (%)	Fat (%)	Fiber (%)	Ash (%)	References
Sudan	Multiple cultivars (e.g., Wad Laqai etc.)	8.78–10.68	78.72–80.41	3.69–4.09	1.71–2.00	2.37–3.14	1.96–2.50	Mohamed et al. (2014)
		—	—	0.11–3.72	0.02–0.33	—	0.23–3.32	Abdel Moneim et al. (2012)
Oman	Fard, Khasab, Khalas	12.6–18.5	77.13–83.41	1.47–1.68	0.52–1.41	6.26–8.0	1.49–1.79	Al‐Farsi et al. (2005)
	Various (e.g., Mabseeli, Um‐sellah, Shahal)	18.77–23.71	—	1.28–1.89	1.24–2.37	1.66–2.38	1.12–1.55	Ali et al. (2009)
		15.00–21.00	74.5–82.4	1.8–3.8	0.1–0.7	1.0–2.5	1.0–2.0	Al‐Harrasi et al. (2014)
Saudi Arabia	Soukari, Barhi, Soughi, Khulas, etc.	1.97–4.82	—	1.51–2.41	—	1.91–3.90	—	Al Juhaimi et al. (2014)
UAE	(e.g., Emirati cultivars)	13.34–20.10	67.33–75.30	1.89–2.73	0.12–0.22	5.52–9.11	1.45–1.89	Habib and Ibrahim (2011)
Tunisia	Various Tunisian cultivars	—	79.93–88.02	0.66–2.85	0.06–0.57	8.09–20.25	1.73–2.59	Borchani et al. (2010)
		—	79.1	2.10	—	14.4	2.50	Elleuch et al. (2008)
Pakistan	Zahidi, Dhaki, Aseel	14.30–17.45	—	—	—	—	1.06–7.87	Khan et al. (2008)
		9.90–14.81	—	2.1–2.7	0.2–0.4	—	1.4–1.9	Anjum et al. (2012)
		6.80–31.90	—	1.9–3.24	0.1–0.46	—	2.00–3.46	Hasnaoui et al. (2012)
		10.50–15.54	—	3.72–4.60	1.28–2.61	4.84–7.19	0.94–1.91	Awan et al. (2018)
Morocco	Eight Moroccan cultivars	13.80	73.00	3.00	2.90	5.20	2.13	El‐Sohaimy and Hafez (2010)

To effectively process date fruit, it is crucial to have a thorough understanding of its physical characteristics. Chemically, date fruit is rich in carbohydrates and dietary fiber, and it also contains proteins, fats, and various vitamins and minerals necessary for optimal physiological function. These components contribute not only to the nutritional value of dates but also to their health benefits, making them an important food source in many cultures (Manickavasagan et al. 2012; Tang et al. 2013).

Date fruit predominantly contains glucose, fructose, and sucrose, with their levels varying according to the variety and ripening stage. Research indicates that lower levels of glucose and fructose are found at the ‘Khalal’ stage, while higher concentrations are present at the ‘Tamr’ stage. In eight Pakistani date palm cultivars, glucose content at the ‘Tamr’ stage ranges from 25.7% to 31.66%, and fructose content varies from 22.48% to 30.58% (Haider et al. 2014). Despite the common perception of dates as a uniform fruit, evidence suggests significant compositional differences in dates grown worldwide and even within the same region (Awan et al. 2018). Despite their high sugar content, dates have a lower glycemic index (GI) compared to apples, apricots, and bananas. This makes them a suitable option for those monitoring their blood sugar levels, as they cause a slower, more controlled rise in blood glucose (Jenkins et al. 2002; Miller et al. 2003). Furthermore, date fruit contains 23 amino acids, with concentrations varying by maturity stage. During the Kimri stage, aspartic acid, glutamic acid, leucine, lysine, serine, and alanine are prevalent, while during the ripening stage, aspartic acid, glutamic acid, leucine, lysine, proline, and glycine are more concentrated (Ishurd et al. 2004).

Dates are low in fat, making them an excellent dietary choice for individuals with dyslipidemia, atherosclerosis, and other cardiovascular conditions. The dominant fatty acids in dates include lauric, myristic, oleic and palmitic acids (Habib et al. 2013; Tang et al. 2013). Additionally, dates are a valuable source of both soluble and insoluble dietary fiber, with content varying between 6.4% and 11.5% across different cultivars (Al‐Shahib and Marshall 2003). Moreover, the high sugar content in dates makes them suitable for developing energy‐dense bars, providing an instant source of energy (El‐Sohaimy and Hafez 2010). Research indicates that the concentration of fiber decreases as the fruit softens during ripening. Enzymatic activity gradually breaks down polysaccharides throughout the ripening process, leading to increased fruit softness and reduced fiber content (Mrabet et al. 2012). These micronutrients play vital roles in various bodily functions, such as oxygen transport, immune function, and enzymatic activities, making dates an important component of a balanced diet (Khan et al. 2008). The diverse health‐promoting effects of date fruit ranging from model to dose and their health outcomes are comprehensively summarized in Table 2.

**TABLE 2:** Date fruit health effects by dose and model.

Study and model	Dose & duration	Health outcomes	Mechanism/biomarkers	References
Humans, T2D RCT	60 g/day fresh dates (3.8 dates twice daily), 12 weeks	No change in HbA1c or fasting glucose; lipid and BP unchanged	Safe glycemic load; no adverse effect on metabolic parameters	Butler et al. (2022)
Humans, T2D RCT	3 dates/day, 16 weeks	↓ Total cholesterol (−0.209 mmol/L), ↓ LDL (−0.171 mmol/L)	Enhanced lipid profile in T2DM patients	Alalwan et al. (2020)
Rats, Hyperlipidemia	300–600 mg/kg/day date suspension, 8 weeks	↓ Triglycerides & VLDL	Improved lipid metabolism	Al‐Dashti et al. (2021)
Rats, Diabetic Cardiomyopathy	5 mg/kg/day extract, w/STZ & HFD, 4 weeks	Cardio protection: reduced cardiac stress markers	Antioxidant, reduced oxidative damage	Nawaz et al. (2024)
Rats, Hyperlipidemic (Triton WR‐1339)	200–400 mg/kg seed extract	↓ Lipid levels	Lipid‐lowering effect in acute model	Hmidani et al. (2020)
Rats, Spathe extract in diabetic rats	Dose unspecified as compared to metformin	↓ Lipid peroxidation and dyslipidemia	Antioxidant, enhances glucose metabolism	Hosseinzadeh et al. (2021)

## 3 Date Preservation and Its Novel Product Formulations

Dates are an exceptionally nutritious food, suitable for consumption both raw and as an ingredient in various value‐added products. Given the substantial production of this fruit, effective preservation and value addition techniques are essential to prevent waste and spoilage. To remain competitive and profitable, food companies continually seek innovative products. It is imperative for a food company to grow and evolve by expanding its range of innovative items or by creating new products from existing ones (Fuller 2016). Within this paradigm, the processing of date fruit necessitates both innovative product development and effective preservation techniques. This dual approach not only prevents waste but also introduces novel methods and valuable date‐based products to the food industry. A variety of date‐based products, their respective processing techniques, and associated nutritional retention findings are summarized in Table 3, offering a comparative insight into their functional potential and industrial relevance.

**TABLE 3:** Date‐based products: processing methods and nutritional retention.

Product	Processing method	Nutritional/bioactive effect	References
Date juice	Thermal pasteurization (80°C, short hold) vs. high‐pressure processing	Pasteurization increased total phenolics and antioxidants more than high‐pressure processing, but high‐pressure processing better preserved proteins and color	Al‐Farisi and Lee (2014)
Date syrup	Ohmic heating (9–11 V/cm) vs. conventional heating	Greater yield; sugars and phenolics retention; less thermal damage at optimized field strength	Al‐Hilphy et al. (2023)
Date syrup	Enzyme‐assisted (pectinase/cellulase) vs. conventional	Enzymatic treatment preserved phenolics and carotenoids better, enhancing antioxidant assay	Abbès et al. (2013)
Date paste	Enrichment with 3% seed powder	+37% dietary fiber, +27% phenolics, improved firmness and antioxidant activity vs. control paste	Al‐Farisi and Lee (2014)

### 3.1 Date Fruit‐Based Liquid Products

Date syrups and essences are produced from low‐quality or industrial‐grade dates. The high sugar content of dates enhances the hygroscopicity and physical appearance of syrups. These syrups can replace sugar in recipes where color is not a primary concern. To produce syrup, dates are mixed with water in a suitable proportion and heated to between 50°C and 60°C for at least 60 min. Additionally, sonication can be employed to improve extraction and ensure microbial inactivation under optimal conditions (Entezari et al. 2004). Earlier, Al‐Farsi (2003) worked on using a modified clarifying procedure to turn low‐quality dates into high‐quality date syrup. Dates, with their high sugar content, are highly susceptible to fermentation under optimal conditions. Throughout history, the fermentation process has been used to produce date wine and vinegar. Various techniques are employed to extract date juice, ranging from simple hot water extraction to sophisticated enzyme‐assisted methods (Peng‐bao et al. 2011). In contrast to traditional hot water and pectinase extraction methods, a previous study found that microwave‐assisted extraction, combined with a higher ethyl acetate concentration, yields a unique aroma and significantly reduces both extraction and fermentation times (Zheng and Lin 2006). The heating impact on date syrup was investigated by (Abbes et al. 2013). The researchers found that boiling the syrup up to 100°C can enhance the types of antioxidants present, thereby increasing antioxidant activity. During heating, the Maillard reaction and caramelization compounds, such as 5‐hydroxymethyl‐2‐furfuraldehyde, which possess strong antioxidant properties, are formed. As a result, date syrup can be added to value‐added food products to boost their natural antioxidant content and promote health.

### 3.2 Date Fruit‐Based Confectionary Products

The developed date paste is an effective and convenient approach to retain the fruit's deliciousness, providing flavorful options for paste incorporation into products (Barreveld 1993). Furthermore, the paste increases relevance in the food industry because it is easier to handle and lowers shipping and storage expenses (Hui 2006). To make the paste, steam cleaned and pitted dates for 3 min at 69 KPa or soak them in hot water (95°C) for 5 to 15 s. After that, the dates are chopped to make date paste. Citric and ascorbic acid are added to avoid changes in color and preserve the desired pH of the final product (Shi et al. 2005). The paste's rheology reflects its viscoelastic properties (Ahmed and Ramaswamy 2006). Date paste is also made with hot water in a 1:1 ratio for 3 min. After manually removing the seeds, the date flesh combination is crushed into a uniform paste. The paste is pasteurized at 80°C after filtering out the blanching water. The investigation of the paste revealed the presence of antioxidants, dietary fiber, and nutraceuticals. The researchers claim that the presence of sugars, particularly reducing sugars, and the neutral pH of the paste makes it acceptable for usage in the confectionery, bakery, and dairy industries (Trigueros and Sendra 2014). In another investigation, second‐grade Deglet Nour dates were utilized to develop paste, which was then used to make date jelly. Fully ripened dates (tamr stage) were washed and then dried at 45°C for 12 h. Following that, the pulp was processed to make a paste, which was then used further (Masmoudi et al. 2010). Date paste is an excellent component that can improve the product's nutritional value while also adding appeal. Date paste is included in bread compositions at a rate of 4%–8%, resulting in significant improvements in the rheological characteristics of date‐based bread. It reduces gelatinization while increasing gas generation and retention efficiency. Furthermore, date paste enhances the overall appearance, crumb, and crust properties. It also improves shelf stability and slows the staling process (Yousif et al. 1991). In a recent investigation, date pit fortified pan bread was made with 5%–10% levels. The inclusion improves the water absorption, stability of dough, dough development, tenderness, and time of the end product (Halaby 2014).

Date paste supplementation in cookies leads to a 20% increase in spread ratio, whereas sugar crystallization is avoided during the cooling process. Date jam and jelly are made from pulp with Brix levels of 65° and 73°, respectively (Shi et al. 2005). Date butter, like peanut butter has been made from completely ripe, high‐sugar dates (Hui 2006). El‐Sharnouby (2012) studied the nutritional value of biscuits enriched with date fruits and wheat bran. Wheat flour was partially substituted with date fruit and wheat bran powder (1:1) at 10%–40% levels. The results showed greater absorption of water with a reduction in dough stability and mixing tolerance index, which could be related to the inclusion of wheat bran.

According to Elleuch et al. (2014) depicted the combination of date fiber concentrate and sesame seed coatings enhances both the nutritional and sensory properties of date halwa. Chopped dates, syrup and paste were used to replace sugar in the traditional Indian meal ‘idli’. The results suggest that date‐containing ‘idli’ has greater vitamin C and polyphenolic levels than regular ‘idli’ (which contains sugar) (Manickavasagan et al. 2013).

Date bars and candies are popular date‐based commodities made with paste and nuts, and they are commonly coated in chocolate for a good sensory quality. High‐energy bars are made with skim milk powder and oat flakes. Ice creams, jams, jellies, and puddings have been produced utilizing date fruit (Awan and Sohail 1999; Besbes et al. 2009). Yet, commercial‐scale processing and technical uses for date fruits have limited possibilities and new options must be investigated. Processing is not on the same scale as other tropical fruits, especially in developing countries.

## 4 Phytochemistry of Date Fruit

**FIGURE 1:**
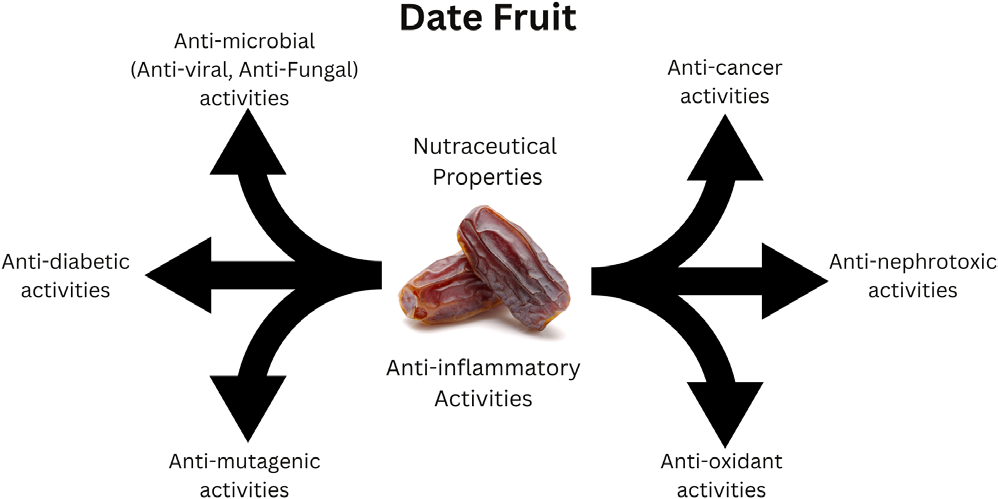
Phytoceuticals in mitigating numerous physiological risks.

Phytoceutical compounds, which are secondary metabolites present in varying levels and types within plant‐based foods, are celebrated for their potential to mitigate various diseases. These bioactive substances contribute significantly to the health‐promoting properties of fruits, vegetables, and other plant‐derived products (Quiñones et al. 2013). Evidence‐based insights highlight the beneficial characteristics of phytoceuticals in mitigating numerous physiological risks, including cardiovascular diseases, renal toxicity, hepatotoxicity, dyslipidemia, hypertension and oncogenesis (Figure 1). These bioactive compounds play a crucial role in various metabolic redox processes by regulating enzyme activity and interacting with diverse cellular pathways. Additionally, high cholesterol intake is a modifiable risk factor associated with oxidative stress‐related issues in vital organs such as the liver, heart, and kidneys (Santilli et al. 2015).

Researchers, healthcare professionals, and medical experts are increasingly focusing on how diet can actively extend life expectancy by regulating various physiological processes and preventing chronic illnesses. In addition, the type and composition of phytochemicals and their consequent antioxidant properties vary depending on the used approach (Chang et al. 2016; Manickavasagan et al. 2012; Rawal et al. 2010; Steinmann and Ganzera 2011).

### 4.1 Extraction of Phytoceutics From Date Fruit

Natural bioactivity encompasses a wide range of structures and functions, offering an enormous molecular pool for the synthesis of natural food additives, designer meals, and nutraceuticals. These compounds are present in varying concentrations in various commodities; frequently, they are present in extremely low levels that need for intensive harvesting in order to achieve sufficient quantities. Under such circumstances, the chemical synthesis is not profitable due to the structural diversity and complexity. Modern extraction technologies have been developed as a result of the inherent challenges in measuring and separating these advantageous components. Conventional solid–liquid or liquid–liquid extraction procedures are among the most widely used extraction techniques; advanced techniques include pressured liquid extraction, ultrasonic and microwave aided extraction methods, and subcritical and supercritical fluid extractions. The commercial use of these technologies can help in the development of individualized nutrition plans to address the growing challenges of physiological hazards (Farag et al. 2014).

The extraction process includes releasing the active ingredients from their corresponding forms in a systematic and sequential manner. Phenolic compounds can be extracted from a variety of samples, depending on the sample matrix type, the molecular structure of the phenolic compounds present, the quantity of hydroxyl and aromatic groups, and the polarity of the polymer. The samples are finely chopped or ground to a small particle size for more effective extraction. Prior defatting of high‐fat samples is necessary to prevent any obstacles during the extraction process. The temperature and duration of the extraction process, the kind of solvent used, the ratio of solvent to sample and the number of extractions performed all affect the yield of phenolics (Al‐Asmari et al. 2017).

The previously listed process conditions are taken into consideration while determining extraction efficiency. When preparing date extract, solvent extraction techniques are typically used. The most common solvents for extracting dates include acetone, methanol, water and ethanol. Dates are blended with a predetermined ratio of solvent and homogenized using an orbital shaker for a few hours; staying overnight is occasionally recommended for optimal extraction. The extracted materials are further subjected to filtration or centrifugation and rotary evaporation is performed to yield the crude extract (Al‐Musayeib et al. 2012; Antolovich et al. 2002; Habib and Ibrahim 2011; Habib et al. 2013; Roginsky and Lissi 2005).

Compared to traditional solvent extraction, supercritical fluid extraction (SFE) is an innovative approach that is thought to be more environmentally friendly. Due to its high diffusivity and selectivity, it is gaining global popularity. The most common supercritical fluid is carbon dioxide, which has a critical temperature of 31.5°C and a pressure of 73 atm. Its low cost, inertness, nontoxicity, and nonflammability make it a good option. The high sugar content of the fruit makes it stickier, which is why this technology is not utilized to extract dates. Nonetheless, the SFE method can be used to extract oil from date seeds. In this case, date seed oil was extracted using SFE (Aris et al. 2013). The maximum oil yield was achieved for 40 min at 41.4 MPa, 70°C, and a 24 mL/min CO_2_ flow rate.

Laurentic, myristic, elaidic, palmitic, capric, and caprylic acids were detected in the obtained oil by GC–MS component analysis. The date seed supercritical extract also included specific phenolic acids, including quercetin, ellagic acid, rutin, and chlorogenic acid. More targeted extraction for particular moieties has been made possible by the rise of nutraceuticals as a disease treatment. This demands the development of a highly sensitive and selective polyphenol detection technique. The existence of structural categories on phenolic substances has led to the development of several spectroscopic techniques. The majority of these are used for total quantification, and relatively few depend on determining their basic components (like anthocyanins and betalains) based on their extinction coefficients. More sophisticated techniques are needed for the screening and measurement of particular polyphenols, though. The most popular, prevailing and commonly utilized method in this regard is HPLC. The extraction process differs depending on the type of moieties to be examined and the equipment being utilized for HPLC quantification of polyphenol determination. For example, acidified methanol is used to extract simple polyphenols from the dates such as hydroxycinnamic acid, monomeric catechins, flavonoids, and procyanidins. Adding up to 1% of acetic or formic acid is the standard method for acidification. In order to achieve better results, the process of depolymerizing procyanidin structures, which transforms procyanidin oligomers into monomeric units, is facilitated by phenolglucinol (Shahidi and Ambigaipalan 2015). The most popular HPLC system consists of a reverse phase C18 column, a solvent system, and a UV–VIS diode array detector. For polyphenol detection, mass spectroscopy and diode array detectors are also employed (El Sohaimy et al. 2015).

### 4.2 Phytochemical Fingerprinting of Date Fruit

Techniques such as High‐Performance Thin‐Layer Chromatography (HPTLC) and High‐Performance Liquid Chromatography (HPLC) are used to obtain chemical fingerprints. These methods, along with hyphenated techniques and spectroscopic methods, are crucial for identifying and evaluating the quality of phytomedicines and medicinal plants. These analytical approaches ensure the accurate assessment of bioactive compounds, aiding in the development of effective and safe natural health products (Rawal et al. 2010; Steinmann and Ganzera 2011). The phytochemical densities in date fruit are detailed in Table 4. Phytochemical fingerprinting analysis is a sophisticated method that provides a detailed and unique profile of the phytoceuticals present in the material. This comprehensive technique allows for the precise identification and quantification of bioactive compounds, ensuring a thorough understanding of the fruit's health‐promoting properties (Ramzan and Li 2015). In addition to these highly precise techniques, other non‐specific methods are frequently employed to quantify the overall content of bioactive compounds. These methods include measuring total phenolics, flavonoids, carotenoid content, and total anthocyanins using pH differential methods. Such techniques are invaluable for providing a broad overview of the phytochemical composition, offering insights into the nutritional and therapeutic potential of date fruits and other plant‐based materials (Chang et al. 2016).

**TABLE 4:** Bioactive compounds in *Phoenix dactylifera* (Echegaray et al. 2021).

Compound	Phytochemicals	Chemical structure	Quantity	Date palm part
Cinnamic Acid	Hydroxycinnamic acids	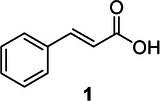	nd‐1.51 mg/100 g FW	Fruit
Sinapic Acid	Hydroxycinnamic acids	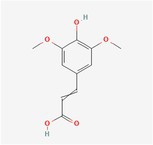	0.09–0.27 mg/100 g FW	Fruit
Ferulic Acid	Hydroxycinnamic acids	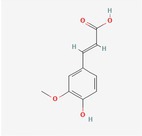	0.31–5.96 mg/100 g FW	Fruit
Vanillic Acid	Hydroxybenzoic acids	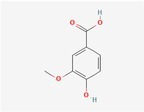	0%–56% of total phenols	Pollen
Syringic Acid	Hydroxybenzoic acids	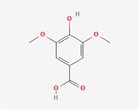	nd‐4.95 mg/100 g FW	Fruit (Dry)
Caffeic Acid	Hydroxycinnamic acids	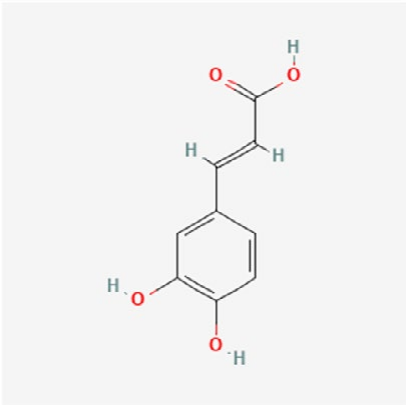	0.18 mg/100 g FW	Seed
Chlorogenic Acid	Hydroxycinnamic acids	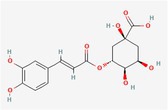	7.11 mg/100 g	Pollen
Coumaric Acid	Hydroxycinnamic acids	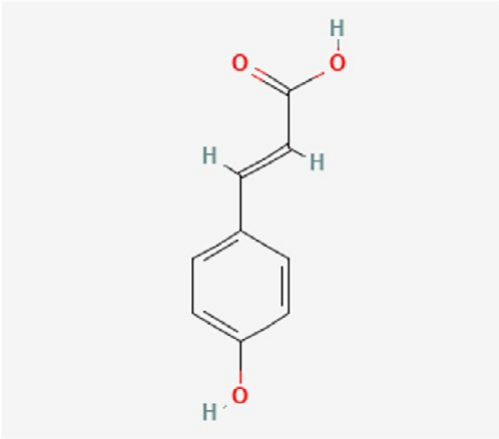	43%–92% of total phenols	Pollen
Procatechuic Acid	Hydroxybenzoic acids	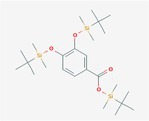	7.9–8.84 mg/100 g FW	Seed
Quercetin	Flavanoids	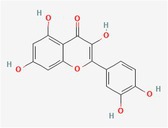	0.17–1.27 mg/100 g DW	Fruit
Luteolin	Flavanoids	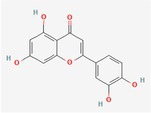	0.02–0.10 mg/100 g DW	Fruit
Methyl quercetin	Flavanoids	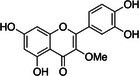	21.93–36.39 mg/100 g	Seed
Methyl luteolin	Flavanoids	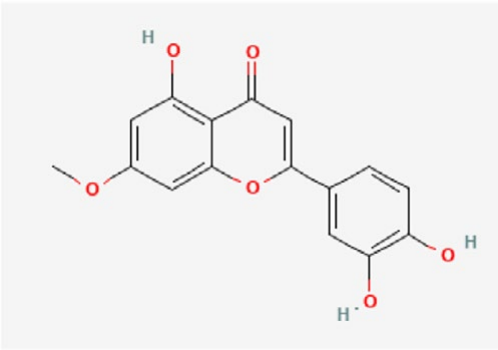	7.62–12.68 mg/100 g	Seed
Catechins	Flavanoids	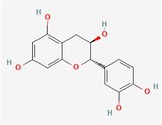	0.32–0.72 mg/100 g	Seed
Epicatechin	Flavanoids	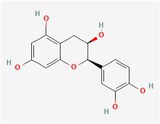	8%–53% of total phenols	Pollen

In addition to these precise methods, certain non‐specific methods are also widely used for overall content quantification. These include the determination of total phenolics and flavonoids, carotenoid content, and total anthocyanins using pH differential methods. However, the non‐specific approaches are typically employed to obtain an overview of the commodity under study's antioxidant capacity. Antioxidant activity is expected to increase due to rising phenolic contents and vice versa.

Twenty‐one Egyptian date varietals' metabolite profiles were previously clarified by Farag et al. (2014). According to the scientists, the total polyphenol content of the chosen date cultivars ranges from 233 to 1897 mg/100 g D.W. Quercetin conjugates and glycosides of luteolin and apigenin were detected by advanced analysis employing UPLC/PDA/ESI‐qTOF‐MS quantification. Among the phenolics, the conjugate of hydroxycinnamic acid, caffeoyl shikimic acid, was found to be the most prevalent. The study examined both aqueous and ethanolic extracts of date fruit, specifically Egyptian dates at the Tamr stage. The outcomes indicated that the ethanolic extract included 10.31 mg GAE/g of total phenolics, while the aqueous extract had a higher content at 14.80 mg GAE/g. According to El Sohaimy et al. (2015), HPLC quantification indicated that tannic acid and aesculetin were found in higher concentrations than gallic, itaconic, and ferulic acids. Four commercial varieties of Saudi dates have been shown to have decreased total phenolic content (76.74–122.20 mg GAE/g). This variation in phenolic content highlights the diverse nutritional profiles of different date cultivars, which can influence their antioxidant capacity and potential health benefits (Al‐Asmari et al. 2017).

Various methods have been employed to characterize the polyphenolic compounds in date fruit. One approach involves using HPLC to directly distinguish procyanidin oligomers from methanolic extracts of date fruit powder. These techniques provide a comprehensive analysis of the polyphenolic content, offering valuable insights into the nourishing and health‐promoting properties of dates (Kennedy and Jones 2001). Hammouda et al. (2013) used the acid depolymerization method to divide the phenolics found in date fruit into five different classes: procyanidin oligomers, flavonoids, flavones, hydroxycinnamic acid and its derivatives, and flavonoids. They discovered that the flesh and peel of the fruit contain about 82% of all the polyphenolic chemicals present in the fruit. Based on the structure of the (−) epicatechins, procyanidins were found to be the most abundant phytoceuticals in Tunisian dates at the Tamr stage. They accounted for around 95.1% of the TP (total phenolics) in the edible part of the dates, at a mediocre degree of about 14 g/kg. Moreover, three Saudi date varieties Barni, Khalas, and Ajwa were tested for polyphenol content, revealing valuable insights into the phenolic profiles and potential health benefits of these cultivars (Benmeddour et al. 2013).

Vanillic, p‐coumaric, isovanillic, hydroxybenzoic, syringic, protocatechuic, caffeic, sinapic, gallic, chlorogenic acids, and ferulic were present in the cultivars. It was found that catechins were among the flavanols. The Ajwa cultivar has the largest concentration of polyphenols and noteworthy anthocyanidins compared to the other two varieties. In 10 Algerian date fruit varieties, one of their colleagues, Benmeddour et al. (2013), found and measured five flavonoids, namely quercetin, quercitrin, luteolin, rutin, and isoquercetrin, along with four phenolic acids, namely gallic, ferulic, coumaric, and caffeic acids. In another investigation, 16 distinct phenolic substances were described using the extract of methanol from four Tunisian date genotypes throughout the maturing process. The most common acids were hydroxycinnamic acids (16.75–19.40 mg/100 g F.W.), followed by ferulic acid (3.48–5.96 mg/100 g F.W.) and caffeic acid (3.04–5.75 mg/100 g F.W.). It was also established how much protocatechuic acid (3.24–4.41 mg/100 g F.W.) and syringic acid (1.33–4.95 mg/100 g F.W.) were produced throughout the “Besser” and “Tamr” stages. However, the Khalat Dhabi variant does not contain any syringic acid. Additionally, a decreased gallic acid content between 0.70 and 2.364 mg/100 g F.W. was detected. With a concentration ranging from 3.34 to 3.84 mg/100 g F.W., the catechin group is the most prevalent among flavonoids (Habib et al. 2013).

Similar to this, Saleh (2011) identified three Saudi date types' hydrophilic antioxidants, which were primarily catechin, rutin, and caffeic acid. For catechin, rutin, and caffeic acid, the concentration ranges are 5.00 to 7.30, 3.60 to 8.10, and 5.40 to 7.40 mg/kg, respectively. Table 5 lists the various stages of phenolic content. Protocatechuic, ferulic, syringic, and vanillic acids are quantified among the free phenolic acids. However, the Omani dates also include bound phenolics, including gallic, p‐hydroxybenzoic, caffeic, p‐coumaric, and o‐coumaric acids. On the other hand, distinct ripening stages at different development levels of date fruit are greatly influenced by bioactive chemicals (Table 3).

**TABLE 5:** Ripening Stages of date and its influence on bioactive compound contents from *Phoenix dactylifera* (Al‐Mssallem et al. 2013).

Development stages		Influence on bioactive compounds	Common bioactive compounds
Hababauk	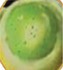	Increases bioactive compounds, after pollination, a whitish‐cream color stage appears 4 weeks later	Nil
Kimri	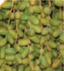	Increases bioactive compounds, Greenish color, hard texture	Nil
Khalal	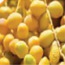	Increases bioactive compounds, Yellowish	Nil
Rutab	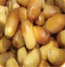	Decreases bioactive compounds, Fruit softens, Sweeter	0.24–1.52 mg/100 g of Anthocyanins 1.31–3.03 mg/100 g of Carotenoids 9.25–42.52 mg/100 g of Phenolic acids
Tamer	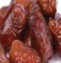	Decreases bioactive compounds, Richest sweetness, velvety texture, and deep brown hue	Carotenoids 0.03–2.9 mg/100 g in *Tamer* Phenolic acids 20.24–64.44 mg/100 g

## 5 Antioxidant Potential & Therapeutic Properties of Date Fruit

### 5.1 In Vitro Antioxidant Properties of Date Fruit

Antioxidant activity is determined by a variety of tests; however, each method has some constraints because the mechanism that measures the effect of antioxidants in regulating the rate of oxidation in intricate multiphase systems cannot directly assess total antioxidant activity. Since no single approach can provide a complete picture, the antioxidant activity of a given extract is explained using a variety of test models (Antolovich et al. 2002; Roginsky and Lissi 2005). Frequently employed techniques for quenching free radicals include the FRAP assay, DPPH technique, ABTS decolorization techniques, hydrogen peroxide scavenging activity, reducing power assay, nitric oxide scavenging capacity, oxygen radical absorbance capacity, and many more. Because it is easy to use and reasonably priced, DPPH is the most popular technique among them. It is a rapid process with minimal steps and chemicals. However, the ABTS assay is thought to be a reliable technique because it may be used with both lipophilic and hydrophilic antioxidants (Alam et al. 2013). These assessments of radical scavenging rely on single electron or hydrogen atom transport processes (Apak et al. 2013; Shahidi and Ambigaipalan 2015). Numerous lines of evidence point to a positive correlation between the concentration of molecules with antioxidant capacity primarily flavonoids and polyphenolic constituents and the potential for antioxidant activity (Amira et al. 2011, 2012; Amorós et al. 2009; Awad et al. 2011; Mansouri et al. 2005). Thus, phenolic and flavonoid concentration measurements without specificity are frequently associated with the samples' potential for antioxidant activity.

The ripening stage of dates significantly impacts their phenolic content. Using methanolic extracts, an investigation assessing 6 Mauritian date cultivars in two edible stages, “Khalal” and “Tamr,” discovered significant variations. Lemine et al. (2014) found that the “Khalal” stage had the highest TEAC capacity, exhibiting a substantial positive correlation between polyphenolic quantity and antioxidant activity. Tunisian date cultivars almost three were found to have significantly different phenolic contents, ranging from 240.38 to 505.49 mg GAE/100 g date extract. These varietal differences also extend to the extracts' radical quenching capacity, as demonstrated by their ability to scavenge DPPH radicals. This variability highlights the influence of cultivar on the antioxidant potential of date extracts. The Allig cultivar has the highest recorded scavenging capacity, at 58.77%. Additionally, three Pakistani date cultivars, Dora, Karbaline, and Dhaki, were tested for antioxidant activity in a research study from Anjum, Bukhat, El‐Ghorab, Khan, Nadeem, Hussain, and Arshad. Of the cultivars evaluated, the Karbaline extract from methanol exhibited the highest level of DPPH radical scavenging activity, with a value of 90.96%. This suggests that it is the most effective antioxidant source Al‐Najada and Mohamed et al. (2014).

Nevertheless, it has been established that Algerian date cultivars have extremely little phenolic contents, ranging from 2.49 to 8.36 mg GAE/100 g F.W. On the other hand, phenolic content and antioxidant activity showed a strong association (Mansouri et al. 2005). The TPC of the date water extract from Ajwa is significantly higher at 455.88 mg/100 g than that of the alcoholic extract, which is 245.66 mg/100 g (Saleh 2011). Moreover, date extract exhibits potential for decreasing the mutagenicity caused by benzo (a) pyrene on Salmonella strains in a dose‐responsive way. Thus, the findings indicated that date fruit contains substances with potent free‐radical‐quenching properties due to its antioxidant and antimutagenic abilities (Vayalil 2002). The antioxidative capacity study of 16 cultivars from Bahrain of altogether ripe dates indicates average fresh weight basis FRAP values of 1.2 mmol/100 g (Allaith 2008).

### 5.2 Restorative Potential of Date Fruit

The chemistry between free radicals and their effects on different diseases have received a great deal of attention years, which leads to enhanced management of diseases and health. There are specific conditions under which oxygen, a vital element for life, poses harmful effects on the human body (Aruoma et al. 2003). Free radicals, mainly, cause disturbance in homeostatic balance at cellular level and leading to the damage of macromolecules (Young and Woodside 2001). The balance between free radicals and antioxidants is necessary to have optimum physiological function. An overabundance of free radicals causes the body to enter an oxidative stress state, causing modification in proteins, lipids, and nucleic acids and can lead to a variety of illnesses (Lobo et al. 2010). One of the main contributors of the disruption of the liver marker enzymes and the correlation between hyperlipidemia and atherogenesis and myocardial infarction is oxidative stress (Lee et al. 2007).

Obesity is considered as one of the prime factors behind this. High‐fat diets or fat accumulation induce oxidative stress, which subsequently causes metabolic problems that are associated with obesity (Furukawa et al. 2017). The remarkable ability of date fruits to prevent disease is largely attributed to their significant antioxidant content and wide array of polyphenolic macromolecules. Traditional medicine systems have been using these preventive qualities since ancient time (Khare 2007; Tahraoui et al. 2007). It is considered beneficial for various skin concerns too as it contains anti‐aging properties. It gives skin a bright and youthful look as it prevents wrinkle formation (Bauza et al. 2001). Pregnant and lactating mothers can also get various benefits from dates consumption. Infants at their teething stage are also fed on date fruits as it is considered to harden the gums (Al‐Kuran et al. 2011; Baliga et al. 2011).

**FIGURE 2:**
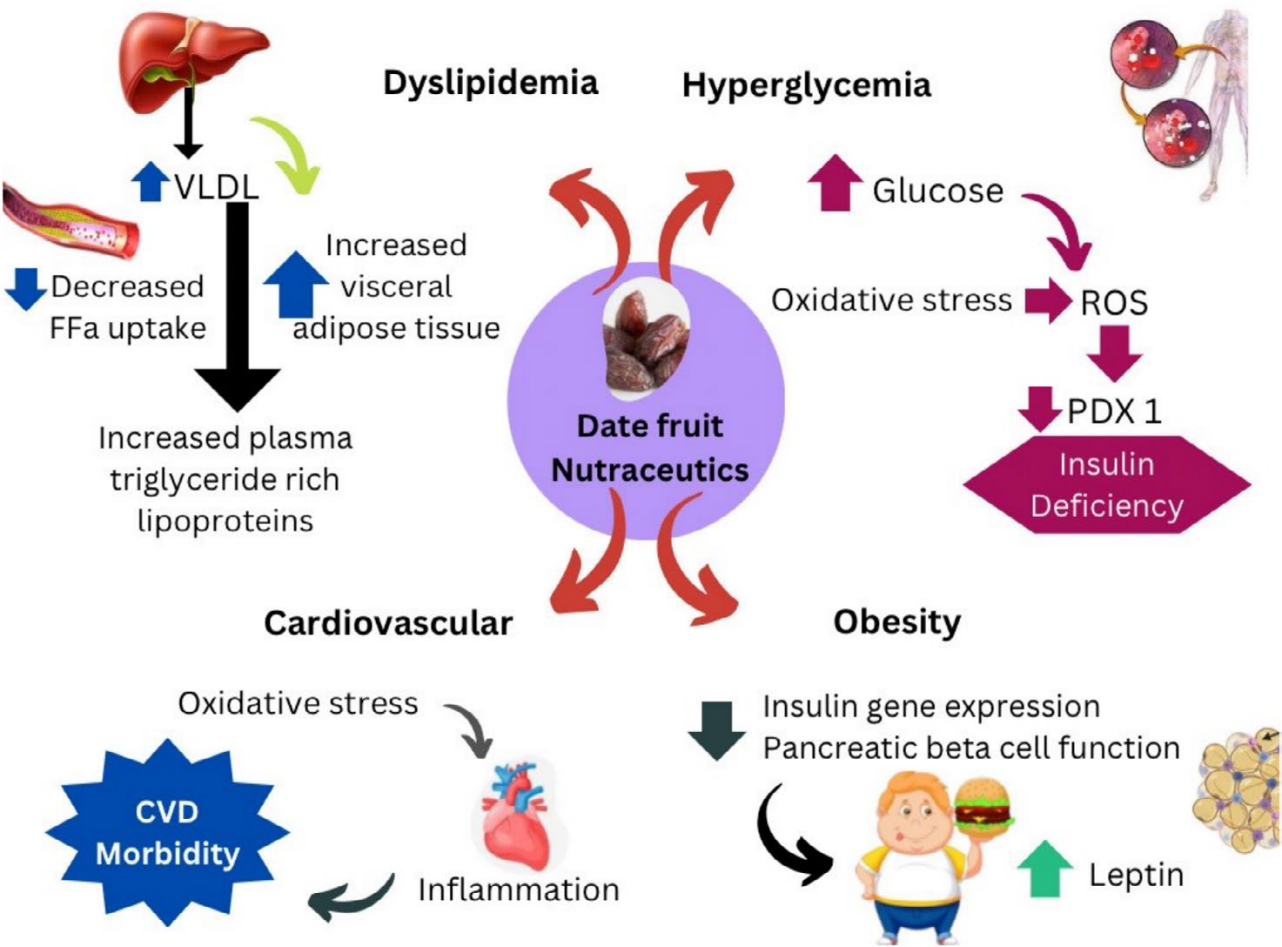
Effects of date phytochemical compounds on different metabolic health complications.

Date fruit extracts have the potential to mitigate the risk of stomach ulcers and also reduce the amount of histamine and gastrin that are produced when ethanol induces ulcers (Al‐Qarawi et al. 2005). Figure 2 demonstrates the effects of date phytochemical compounds on different metabolic health complications, including, cardiovascular health, hyperglycemia, obesity, and dyslipidemia. The pathways illustrate the potential benefits of date fruit components in enhancing insulin gene expression, pancreatic beta cell function, leptin control, oxidative stress, inflammation, lipid profiles, and overall cardiovascular morbidity. The visual representation highlights the date fruit's broad advantages in treating metabolic problems through several processes.

**FIGURE 3:**
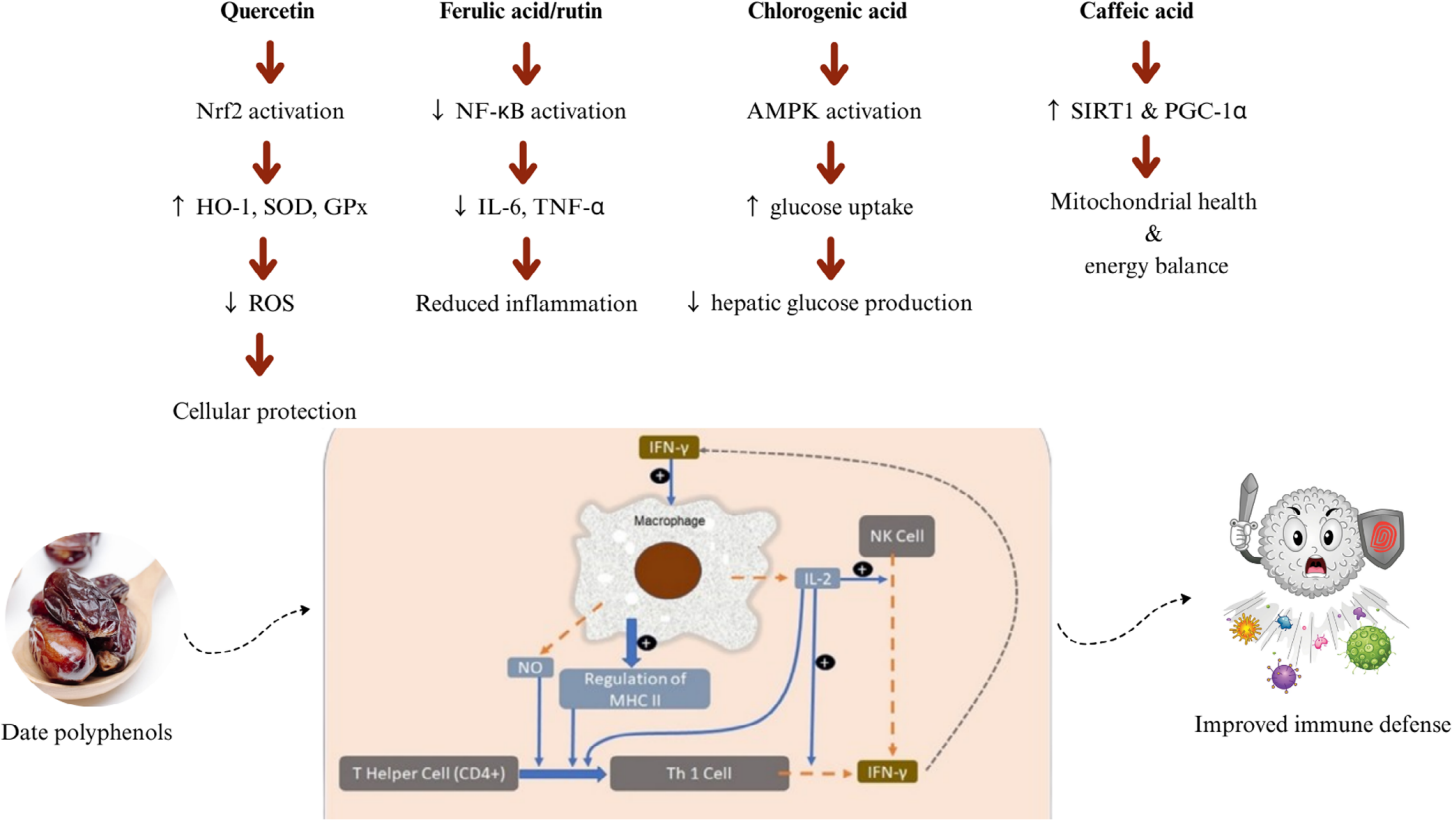
Mechanistic insights into bioactive compounds of date fruit.

Significant neuroprotection against free radical‐mediated oxidative stress, memory impairment, neuronal damage, and spatial learning has been shown by the methanolic date extracts (Pujari et al. 2014). Additionally, date fruit extract improves the pathological markers of diabetic nephropathy in rats and hinders the progression of diabetes (Zangiabadi et al. 2011). The polyphenols found in date fruit, specifically the caffeic, chlorogenic, pelargonin, and ferulic acids, significantly elevate the expression of IFN‐γ mRNA in mice. Peyer's patch cell cultures show that this fruit can activate mice's cellular immune system (Karasawa et al. 2011). Several studies authenticate the anti‐inflammatory antibacterial, immunomodulatory, hepatoprotective and nephroprotective activity of date fruits (Ali et al. 2012; Tang et al. 2013). The necessity of studying natural compounds' potential as preventative agents is growing as the number of disorders in society rises. Dates' ability to protect the heart, liver, and kidney from oxidative stress has been well examined. Depending on the literature that is currently available, the data have been divided into three sections. In order to get at a more definitive conclusion on the molecular targets of date fruit, the total effect of the fruit and the effects of its primary bioactive are detailed in Figure 3. The underlying disease and the circumstances that led to the stress have been covered in the following sections.

### 5.3 Cardioprotective Aspects of Date Fruit

Cardiovascular diseases are the major cause of mortality globally. Several risk factors contribute to the development of these complications. Non‐changeable risk factors include genetic inheritance, age, and gender. Modifiable risk factors include excessive use of alcohol, poor physical activity, and improper diets. Therefore, improving lifestyle and behavior can significantly reduce the risk of prevalent cardiac conditions (Guo et al. 2016). Scientific studies have established a strong connection between oxidative stress and accelerated atherosclerosis, which significantly contributes to the increase in cardiac disorders. The process begins with the damage to the vascular endothelium caused by metabolic conditions such as hypercholesterolemia. Reactive oxygen species (ROS) are now believed to play a key role in the development of atherosclerosis. The identification, aiming, and decrease of lifestyle factors is undoubtedly the utmost potential for decreasing cardiac impediments along with allied ailments (Deepa and Varalakshmi 2003; Elahi et al. 2009). Numerous studies have emphasized that consuming a diet rich in antioxidants can help mitigate oxidative stress and reduce the risk of atherosclerosis caused by lipid abnormalities (Zhong et al. 2015). In this regard, date fruit contains specific bioactive compounds that contribute to the ability to restore heart function.

The hypolipidemic effect of dates is observed by oral administration of date fruit extract on high‐fat diet induced rats which changes distressed lipid profile to normal level (Vembu et al. 2012).

Rock et al. (2009) examined the influence of date (Medjool and Hallawi) consumption on blood lipid and glucose profiles and also determined the oxidative stress markers in healthy subjects. Dates at 100 g/day were administered to 10 healthy subjects for a period of 4 weeks. It was found that body mass index and cholesterol levels do not significantly affect date consumption. For the glycemic profile, the dates, being sweet in nature, are not considered appropriate as no increase was observed in the blood glucose level. The ingestion of Medjool and Hallawi dates considerably decreases the triacylglycerol levels in serum by up to 15% and 8%, respectively. A substantial reduction of 33% in basal serum oxidative stress and 12% in lipid peroxidation has been documented. The authors concluded that dates can be considered an anti‐atherogenic agent. Similarly, the effect of date supplementation on lipid profiles was studied in hypercholesterolemic hamsters as studied by Alsaif et al. (2007). Body weight in normal and hypercholesterolemic hamsters decreases from 38.78 to 35.24 g after 13 weeks of supplementation and from 48.75 to 39.13 g in hypercholesterolemic groups. The ingestion of date fruit also lowers the serum lipid indicators. In the normal group, the level of cholesterol was noted dropping from 109.70 to 97.30 mg/dL, but in the group of cholesterolemic individuals, the high cholesterol diet causes the blood cholesterol level to rise to 309.90 mg/dL, which is then reduced by eating dates (240.80 mg/dL). In a similar vein, the normal group's triglyceride and LDL levels drop sharply from 95.90 to 68.40 and 16.90 to 10.60 mg/dL, respectively. A noteworthy decrease was noted in the hypercholesterolemic group; the date fruit‐containing diet caused the triglyceride level in the high cholesterol diet group to drop from 210.90 to 142.90 mg/dL. Within the hypercholesterolemic group fed a fruit‐based diet containing dates, the LDL drops from 61.50 to 27.40 mg/dL. Significant elevation of the HDL level is also noted. Moreover, Rosenblat et al. (2015) clarified how date and pomegranate polyphenols interact with Eo mice. Amari and Hallawi, two date varietals, were examined. The administration of pomegranate extract and date fruit and seed extract for 3 weeks resulted in a significant reduction in serum triglyceride and cholesterol levels. Additionally, there was an increase in serum paraoxonase (PON1) activity, which is protective against lipid oxidation and prevents the buildup of lipoperoxides in low‐density lipoproteins. Awan et al. (2019) assessed the impact of date fruit and extract on the serum lipid profile of rats that were subjected to oxidative stress. In rats on a normal diet, the authors observed a 3.65% reduction in serum cholesterol; in rats under atherogenic stress, the reduction was 15.14%. However, in stressed and normal rats, the date extract treatment decreased serum cholesterol by 18.55% and 4.49%, respectively. As a result, both fruit and extract contribute to improving the serum lipidemic profile by reducing serum cholesterol. Foods enhanced with polyphenols or flavonoids and CVDs are inversely correlated, according to a number of prospective cohort studies. Numerous intervention studies have shown that these meals have protective effects on intermediate indicators of heart disease.

Al‐Yahya et al. (2016) examined the cardioprotective, anti‐inflammatory, and lipid‐lowering capabilities in a different investigation. According to the authors, lyophilized Ajwa date extract increases H9C2 proliferation by 40% and attenuates cytotoxicity. Furthermore, lipid peroxidation levels are reduced and endogenous enzyme activity (SOD, CAT, and NO) is restored by oral administration of the extract at 250 and 500 mg/kg B.W. Similarly, it suppressed the expression of the proinflammatory cytokines and anti‐apoptotic protein. Ajwa date extract has been shown in histoarchitectural investigations to have a preventive impact by reducing necrosis, inflammatory cell infiltration, and edema. There is also a notable improvement in the biomarkers for cardiac stress and lipidemia. Thus, date fruit may have a therapeutic effect in treating cardiac dysfunctions, particularly those involving disruptions in lipoprotein metabolism. In Sprague Dawley rats fed an atherogenic diet, Awan et al. (2019) examined the effects of date fruit and date extract on oxidative stress and lipid profile. A normal diet control group (G_1_), date fruit and normal diet (G_2_), date extract and normal diet (G_3_), atherogenic diet (G_4_), date fruit and atherogenic diet (G_5_), and date extract and atherogenic diet (G_6_) were the six groups into which the rats were divided by the researchers. This study's findings are quite encouraging. Supplementing with date fruit decreased serum cholesterol levels in atherogenic rats by 15.14% and in normal rats by 3.65%. Similarly, in atherogenic and normal rats, date extract administration resulted in decreases of 18.55% and 4.49%, respectively. When date fruit and extract were given to normal rats, low‐density lipoprotein (LDL) cholesterol, a major risk factor for cardiovascular disease, dropped by 8.66% and 11.55%. However, more significant drops of 21.05% and 25.98% were seen in the atherogenic groups that also received date fruit and extract supplements. Based on blood lipid data, the researchers computed multiple atherogenic ratios to evaluate the effect on cardiac risk in more detail. The atherogenic diet groups showed higher risk than the normal groups, as was to be predicted. Date fruit and extract‐containing functional meals, on the other hand, successfully reduced the elevated risk ratios, indicating their potential to reduce the risk of cardiovascular disease.

Borochov‐Neori et al. (2015) studied the anti‐atherogenic properties of date fruit's polyphenolic moieties on two cultivars: Amari and Hallawi. The examined date extracts differ in their capacity to suppress LDL oxidation as well as in their range of radical scavenging characteristics (Zhong et al. 2015).

Date fruit contains a significant amount of quercetin Farag et al. (2014), and evidence‐based insights point to its potential cardioprotective benefits. In this sense, 4‐ and 12‐week‐old rats were used to test the effects of quercetin therapy. After 4 weeks of treatment at a dose of 20 mg/kg/day, young rats showed signs of both contraction and relaxation indicators, as well as post‐ischemic recovery of left ventricular developed pressure, in response to quercetin. However, in the case of adult rats, no substantial effect was observed (Bartekova et al. 2016). Additionally, it has been proposed that quercetin initiates the cardio prophylaxis against oxidative stress‐induced cell death; yet, extended exposure can potentially result in cardiotoxicity (Daubney et al. 2015). Additionally, rutin offers significant protection against improved ECG parameters, diabetes‐associated oxidative stress, and prevents degenerative changes in cardiac tissues (Saklani et al. 2016).

Numerous flavonoids' effectiveness has been determined and investigated. Numerous date fruit cultivars contain the phytoestrogen kaempferol (Eid et al. 2013). Certain anti‐inflammatory and anti‐apoptotic effects are exhibited by kaempferol. Research has shown an opposite relationship between kaempferol intake and heart conditions (Bhouri et al. 2011; Jang et al. 2011; Kim et al. 2010; Lin et al. 2007). To study the preventive effect of kaempferol against doxorubicin‐induced cardiotoxicity, Xiao et al. (2012) conducted research in this area. The restorative potential of kaempferol in reducing myocardial ischemia/reperfusion injury in rat hearts was investigated by Zhou et al. (2015). Rats treated with kaempferol show a notable improvement in heart functioning. Moreover, it lessens the injury‐induced apoptosis.

The acid phenol, in addition to its ability to reduce oxidative damage in doxorubicin‐induced cardiac damage, p‐coumaric acid is a precursor to other phenolic substances. When stressed mice are given 100 mg/kg of p‐coumaric acid for 5 days, their CPK and LDH enzyme activities are ameliorated by 39.65% and 32.89%, respectively. Furthermore, p‐coumaric acid therapy increases the lowered superoxide dismutase and catalase activity in stress‐induced rats by 41.13% and 15.44%, respectively Abdel‐Wahab et al. (2003) Gallic acid Amira et al. (2012), a metabolite of propyl gallate, may also contribute to the cardioprotective qualities of date fruit. Based on available data, gallate compounds' capacity to bind to lipid membranes is thought to be the primary determinant of their antioxidative effects (Shahrzad et al. 2001). Rats treated with ISO were given 7.5 and 15 mg/kg of gallic acid in a study by Priscilla and Prince (2009). Following a 10‐day investigation, it was found that higher levels of creatine kinase (CK), lactate dehydrogenase (LDH), and creatine kinase‐MB (CK‐MB) are reduced by gallic acid at a dose rate of 15 mg/kg. Hsu and Yen (2007) also looked at the impact of gallic acid on oxidative stress, obesity, and dyslipidemia. It was a high‐fat diet that was used to create obesity. Rats fed a normal and high‐fat diet were assessed at two different gallic acid concentrations: 0.1% and 0.2%. Rats fed a high‐fat diet show a considerable reduction in body weight after receiving gallic acid therapy. In obese rats administered gallic acid, there is also a significant decrease in the serum levels of triacylglycerol and LDL, which significantly improves the circumstances of hepatic steatosis. Additionally, gallic acid and the immunosuppressive medication cyclosporine A together increase cell membrane integrity against ischemia/reperfusion oxidative stress, mitochondrial preservation, and cardiac marker enzymes (Badavi et al. 2014). Gallic acid may therefore be essential for lipid metabolism and the mitigation of oxidative stress. In addition to the aforementioned polyphenolic components, the date fruit's nutritional fiber, vitamins, and minerals may also have a role in its potential for cardioprotection. To gain a deeper knowledge of date fruit's potential to reduce heart stress, more thorough research is necessary.

### 5.4 Hepatoprotective Ability of Date Fruit

The liver is essential to the metabolism of xenobiotics. To be metabolized, the medications and other substances must be delivered to the hepatic system. These substances frequently undergo biotransformation into water‐soluble moieties through different Phase‐I and II detoxification processes. They are removed by urine after being discharged from the liver into the bile or the circulation (Mantena et al. 2008). Bastway et al. (2008) assessed the hepatoprotective effect of aqueous date extracts on thioacetamide‐induced hepatotoxicity in rats. In normal rats, serum alkaline phosphatase (ALP) activity was 60.04 IU/L; in hepatotoxic rats, it rose to 125.38 IU/L. In the hepatotoxic group, the date fruit extract therapy at 4 mL/kg body weight causes a significant drop in ALP, up to 88.03 IU/L. According to Bastway et al. (2008), the activities of aspartate amino transferase (AST) and alanine amino transferase (ALT) also decrease from 117.73 to 62.50 and 78.38 to 48.39 IU/L, respectively. On the other hand, hepatic enzymes increased by thioacetamide and the concentration of plasma bilirubin are dramatically reduced by aqueous date extract.

By lowering hepatic TBARS (thiobarbituric acid reactive substances) levels, restoring hepatic enzyme function, and reducing hepatic DNA fragmentation, date fruit extracts mitigate chemically induced liver stress (El Arem et al. 2014). Male Wistar rats were given aqueous date extract (4 mL/kg) for 2 months while they were fed dichloroacetic acid (0.5 and 2 g/L). The outcomes demonstrated the fruit extract's capability for restoring serum biochemical parameters. For example, the hazardous group fed on dichloroacetic acid at 0.5 and 2 g/L had reported AST activities of 124.50 and 157.72 IU/L, respectively. When aqueous date extracts are administered, the AST indices drop to 115.00 and 132.75 IU/L, respectively. Comparably, in the harmful group induced by 0.5 g/L dichloroacetic acid, the ALT level drops from 60.81 to 57.31 IU/L, whereas in the other group, it drops from 68.75 to 62.75 IU/L. The group fed on date extract with a greater dose of dichloroacetic acid showed the greatest decrease in both γ GT and LDH levels, with values ranging from 56.67 to 39.31 and 734.62 to 686.01 IU/L, respectively. When DCA was used to treat lipid peroxidation, a dose‐dependent increase was seen. On the other hand, treatment with aqueous date extract reduces lipid peroxidation and increases the levels of liver antioxidant enzymes (SOD, CAT, and GPx). The hepatotoxic groups have significant changes in the hepatic histoarchitecture, whereas the normal groups display normal structure. In the hepatotoxic groups, aqueous date extract therapy improves the altered liver histology. In the dimethoate‐intoxicated group, the histological tests show substantial alterations involving congestion, hepatic sinusoids enlarging, mononuclear cell infiltration, and hepatocellular destruction. On the other hand, the pretreatment of date fruit in the dimethoate‐induced hepatotoxic group improves the overall morphology of the liver; mild inflammation is noted, but the lack of vacuolization and necrotic cells indicates that the date fruit extract has the ability to ameliorate the effects on the hepatic tissues (Saafi et al. 2011).

The most opulent dates are those that are cultivated in Madinah, Saudi Arabia, namely the Ajwa variety. Sheikh et al. (2014) examined the hepatoprotective properties of Ajwa dates in male Wistar rats. Rats were given a purposeful dose of 1 g/kg/body weight of date extract, which is equivalent to 7 dates per human per day. Rats that were made hepatotoxic by CCL4 were used to test the effects of date extract for 4 and 12 weeks. The results show that as compared to the control hepatotoxic groups, the date fruit fed groups had significantly lower serum AST and ALT values. In male Wistar rats, the effects of date fruit on biochemical and histological changes induced by lambda cyhalothrin (LTC) were investigated by Ramadhas et al. (2014).

The hepatoprotective qualities of date fruit's catechin and quercetin are also well‐established. The restorative activity of these two compounds was investigated against chlorpyrifos‐induced hepatotoxicity in rats in a study by Uzun and Kalender (2013). Following the 4‐week trial, the hepatotoxic group experienced a 76% increase in lipid peroxidation levels. However, groups treated with quercetin and catechin showed, respectively, a 33% and 31% decrease in lipid peroxidation. The hepatotoxic groups experienced a statistically significant decrease in ALP, AST, ALT, and LDH activities while receiving quercetin (14%, 17%, 19%, and 12%, respectively) and catechin (15%, 28%, 18%, and 14%, respectively). Ramesh et al. (2009) assessed the protective effect of epigallocatechin gallate against hepatic oxidative damage generated by atherogenic diet. The hepatic antioxidant enzyme activities that are restored in rats fed green tea catechins are dramatically reduced by an atherogenic diet. Atherogenic diet‐fed rats have been found to have higher serum levels of AST, ALT, ALP, and LDH activities. The primary green tea catechin, epigallocatechin gallate, which is also a crucial component of date fruits, is primarily responsible for the hepatoprotective qualities of the examined green tea catechins.

### 5.5 Reno‐Protective Potential of Date Fruit

The kidney is a vital component of the urinary system that is mainly responsible for blood pressure regulation, electrolyte balance maintenance, fluid homeostasis, and blood purification and waste disposal. The renal system filters about 180 L of blood every day (Awan 2011). However, exposure to hazardous substances that cause nephrotoxicity puts the kidneys at risk for damage. In the developing world, one of the main causes of illness and death is kidney disease. Renal failure is thought to be the ninth most common cause of death worldwide (Negi and Mirza 2020). Reactive species are produced, and oxidation brought on by excessive nutrient ingestion is frequently the root cause of renal problems. El Arem et al. (2017) clarified the preventive function of date fruit against renal injury. In mouse experimental modeling, El‐Mousalamy et al. (2016) clarified the impact of date fruit extract, both aqueous and methanolic, on diabetic nephropathy. The results demonstrated the reno‐protective benefits of date extracts by enhancing antioxidant and lipid and glucose homeostasis. The body weight of the diabetes control group rises to 248.34 g, while the body weight of the methanolic and aqueous date extract groups decreases to 194.60 and 196.29 g, respectively. The diabetic group's fasting blood glucose level was 464.71 mg/dL; in the aqueous and methanolic extract given groups, it was 125 and 154.71 mg/dL, respectively. Along with a large increase in HDL, the researchers also saw a significant drop in the increased lipidemic indicators, such as cholesterol, triglycerides, and LDL. The application of aqueous date extract lowers serum creatinine from 2.92 to 1.44 mg/dL on therapy. Similarly, in the diabetic control group, the urea level was 71.57 mg/dL; in the rats treated with aqueous date extract, this value drops to 34.14 mg/dL. According to their research, aqueous date fruit extract has greater ameliorative potential than methanolic date fruit extract.

Date fruit is enriched with vitamin C and it possesses qualifying potential contrary to renal dysfunctions as evident from research studies (Das and Büchner 2007; Karabulut‐Bulan et al. 2008). Date fruit also holds quercetin in considerable amounts (Tang et al. 2013). The nephroprotective influence of quercetin is evident from several studies. It is supposed that quercetin retains certain vasodilator properties that cause increased renal blood flow (Gaeta 2018). The protective role of quercetin against fluoride‐induced nephrotic damage was studied by some authors (Nabavi et al. 2012).

## 6 Conclusions

The present review provides a thorough overview of the nutritional composition and bioactive compounds found in date fruit, highlighting their health benefits for humans. Additionally, it explores the incorporation of date fruit into various food products. Dates and their phytochemical constituents possess significant functional and prophylactic properties that can help manage several metabolic disorders, including cardiovascular diseases, liver toxicity, kidney toxicity, hypertension, dyslipidemia, and cancer. Scientific evidence supports the effectiveness of phytochemicals in dates in alleviating oxidative stress‐related dysfunctions. Aqueous extracts of dates have demonstrated hepatoprotective properties, significantly lowering plasma bilirubin levels and hepatic enzymes. Furthermore, date extracts have shown protective effects against nephrotoxicity, attributed to their antioxidant, anti‐apoptotic, and anti‐inflammatory properties, which contribute to renal protection. The presence of polyphenols, dietary fiber, vitamins, and minerals in date fruit may also enhance its cardioprotective effects. However, further in‐depth studies are necessary to fully understand the role of date fruit in reducing cardiac stress. Given that date fruit is cultivated globally, it presents opportunities for the creation of nutritious, value‐added products that offer numerous health benefits. Extensive research has already been conducted on its health‐enhancing effects; thus, future investigations should focus on elucidating the mechanisms behind the functionality of bioactive compounds and their health benefits across different varieties of date fruit. In conclusion, the extensive nutraceutical potential of date fruit and its bioactive compounds holds promise for the development of functional foods aimed at preventing and managing various health conditions. The global cultivation and availability of dates provide a valuable resource for the food industry to innovate and create health‐promoting products. Continued research into the specific mechanisms of action of date‐derived phytochemicals will further our understanding and utilization of this fruit in both clinical and dietary applications, paving the way for enhanced human health and well‐being.

## Author Contributions

**Kanza Aziz Awan: conceptualization (equal), data curation (equal), methodology (equal), visualization (equal), writing – original draft (equal). Sanabil Yaqoob:** conceptualization (equal), data curation (equal), methodology (equal), visualization (equal), writing – original draft (equal). **Iahtisham ul‐Haq:** data curation (equal), methodology (equal), writing – review and editing (equal). **Hiba Naveed:** Data curation, methodology, investigation, writing‐review and editing. **Aysha Imtiaz:** conceptualization (equal), data curation (equal), investigation, visualization (equal), writing – review and editing (equal). **Amal Shaukat:** software, methodology, writing‐review and editing. **Waleed Sultan:** data curation (equal), investigation (equal), methodology (equal), writing – review and editing (equal). **Jian‐Ya Qian:** conceptualization (equal), investigation (equal), project administration (equal), writing – review and editing (equal). **Esther Ugo Alum:** supervision, funding, project administration, visualization, writing‐review and editing. **Qing Shen:** conceptualization (equal), methodology (equal), project administration (equal), visualization (equal), writing – review and editing (equal).

## Conflicts of Interest

The authors declare no conflicts of interest.

## Data Availability

All the authors declare that if more data is required, then the data will be provided on a request basis.
